# Institutional Needs

**Published:** 1920-05-29

**Authors:** 


### Institutional Needs.

THE LYXHAYR MATTRESS.

The hard wear and special purpose to which hospital bedding is exposed make the composition of the stuffing a matter of great importance. It must be able to stand incessant pressure without becoming bumpy; it must not deteriorate, even when very drastically disinfected; it must not disintegrate when remade. Careful tests have been made on Lyxhayr by more than one competent authority in these directions, and it has come well out of them all. It is a purely vegetable material, thoroughly sanitary, and a great improvement on any animal substance used for the same purpose. Wherever introduced it comes to stay.

